# Lipid Production in *Nannochloropsis gaditana* during Nitrogen Starvation

**DOI:** 10.3390/biology8010005

**Published:** 2019-01-08

**Authors:** Jorijn H. Janssen, René H. Wijffels, Maria J. Barbosa

**Affiliations:** 1 Bioprocess Engineering, AlgaePARC, Wageningen University and Research, P.O. Box 16, 6700 AA Wageningen, The Netherlands; rene.wijffels@wur.nl (R.H.W.); maria.barbosa@wur.nl (M.J.B.); 2 Faculty of Biosciences and Aquaculture, Nord University, N-8049 Bodø, Norway; 3 Department of Biology, University of Bergen, P.O. Box 7803, 5006 Bergen, Norway

**Keywords:** *Nannochloropsis gaditana*, lipid production, nitrogen starvation, biomass-specific photon supply rate, triacylglycerol, eicosapentaenoic acid

## Abstract

The microalga *Nannochloropsis gaditana* is a natural producer of triacylglycerol (TAG) and the omega-3 fatty acid eicosapentaenoic acid (EPA). TAG accumulation is induced by nitrogen starvation. The biomass-specific photon supply rate used had an effect on EPA and TAG accumulation during nitrogen starvation as well as on the localization of EPA accumulation. Clear differences in TAG yield on light were found for different biomass-specific photon supply rates and light regimes during nitrogen starvation. De novo EPA synthesis or the translocation of EPA between lipid fractions might be limiting for EPA accumulation in TAG. Further studies are needed to fully understand EPA accumulation in TAG during nitrogen starvation. To elucidate the function of EPA in TAG nitrogen recovery, experiments are suggested. The overexpression of genes involved in de novo EPA synthesis and translocation is proposed to elucidate the exact metabolic routes involved in these processes during nitrogen starvation. This work addresses future opportunities to increase EPA accumulation.

## 1. Introduction

Microalgae are known for their ability to produce large amounts of lipids, and some species are the primary producers of the omega-3 fatty acid eicosapentaenoic acid (EPA). However, this fatty acid is present in small concentrations in the cells; there is up to 4.3% EPA on a dry weight basis in *Nannochloropsis gaditana* [1]. Higher values are required to make this process competitive.

*Nannochloropsis gaditana* is a microalga species known for its capacity to accumulate large amounts of triacylglycerol (TAG) and to produce EPA. One of the most commonly used strategies to induce TAG accumulation is nitrogen starvation. This strategy is often applied as a two-phase batch cultivation, starting with a growth phase and followed by nitrogen starvation, in which TAG is produced de novo and by the conversion of membrane lipids [2]. The acyl–CoA-dependent pathway is used for the de novo synthesis of TAG [3], while polar membrane lipids can be converted to TAG via the acyl–CoA-independent pathway. In general, TAG contains higher amounts of saturated fatty acids and membranes contain higher amounts of polyunsaturated fatty acids (PUFAs), like EPA in some microalgal species [4]. During nitrogen starvation, however, EPA accumulation in TAG increases [5,6]. It is hypothesized that PUFAs are stored in TAG during nitrogen starvation to allow for rapid incorporation into plastid membranes upon more favourable growth conditions [7]. Previously, translocation of intact EPA from the polar lipids to TAG was proven using labelled carbon [8]. In addition to the translocation, EPA was also de novo synthesized during nitrogen starvation.

To be able to increase the EPA concentration in TAG, a better understanding of the accumulation mechanisms and its regulation are necessary [9], alongside knowledge of the impact of the cultivation conditions. Different biomass-specific photon supply rates during nitrogen starvation tested for *Chromochloris zofingiensis* showed no large differences in TAG yield on light [10]. Despite light being the most important and often limiting substrate in microalgae photosynthetic processes, the effects of the biomass-specific photon supply rates and supply mode on TAG and EPA accumulation during nitrogen starvation for *Nannochloropsis gaditana* were unknown.

Different studies describe experiments using various light intensities and initial biomass concentrations at the onset of nitrogen starvation, resulting in various initial average biomass-specific photon supply rates. In addition, the light regime (day:night cycle and continuous light) is often varied. The biomass-specific photon supply rate was shown to influence TAG and EPA accumulation [11]. The effect of a wide range of conditions on EPA and TAG accumulation during nitrogen starvation can bring more insight into the mechanisms involved, and allow the identification of limiting steps on TAG and EPA accumulation, and ultimately its improvement. 

**Figure 1:**
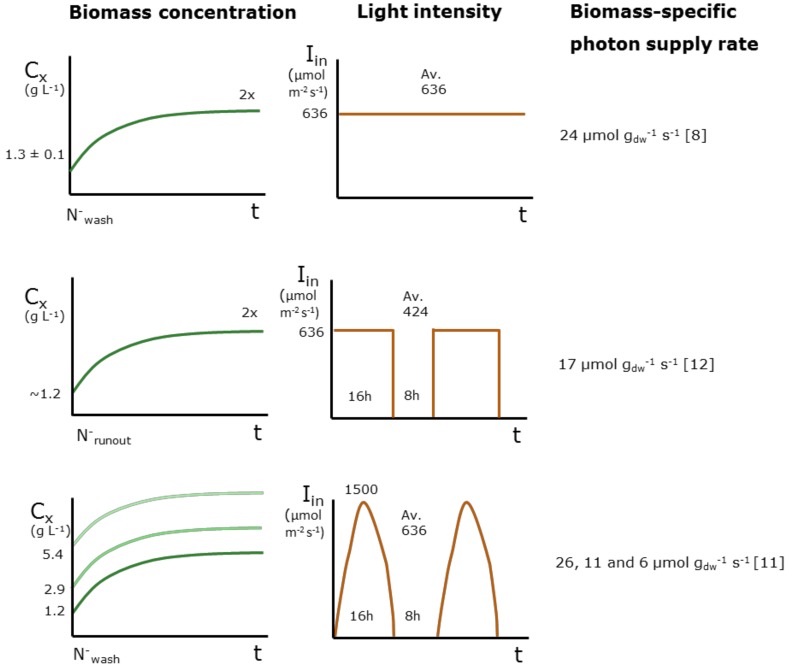
Overview experimental conditions used; light regime, average light intensity per day (µmol m^−2^ s^−1^) and biomass concentration (g L^−1^) at the onset of nitrogen starvation with their respective average initial biomass-specific photon supply rates (µmol g_dw_^−1^ s^−1^) at the start of nitrogen starvation [8,11,12].

In different studies, *Nannochloropsis gaditana* was grown in an airlift-loop, flat-panel photobioreactor with a reactor depth of 20.7 mm at temperature (26 °C) and pH (7.5). The incident light intensity, light regime and initial biomass concentration were all different, resulting in different initial average biomass-specific photon supply rates [8,11,12]. Figure 1 shows the different average biomass-specific photon supply rate at the start of nitrogen starvation. The fatty acid accumulation in TAG and polar lipids was measured. The average incident light intensity used was the same, except for [12] (Figure 1). The biomass-specific photon supply rates of [8,11] were similar, 24 and 26 µmol g_dw_^−1^ s^−1^ respectively, but the light regime was different. In [11], light was supplied as a day:night cycle in a half sinus function instead of a continuous function.

## 2. Triacylglycerol (TAG)

### 2.1. Time-Averaged TAG Yield on Light

**Figure 2:**
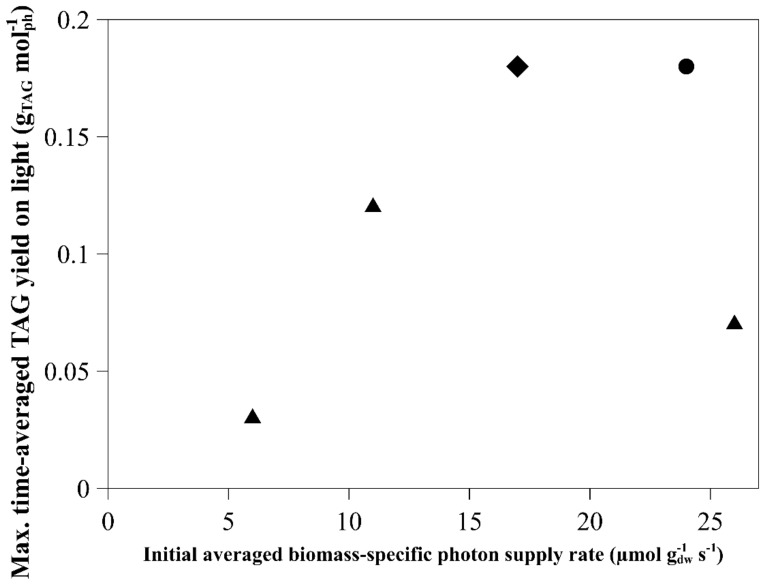
Maximal time-averaged triacylglycerol (TAG) yield on light (g_TAG_ mol_ph_^−1^) reached in the experiments at the initial averaged biomass-specific photon supply rates as shown in Figure 1. The light regime used: continuous (circles), sinus (triangles) and block (diamonds) [8,11,12].

**Figure A1:**
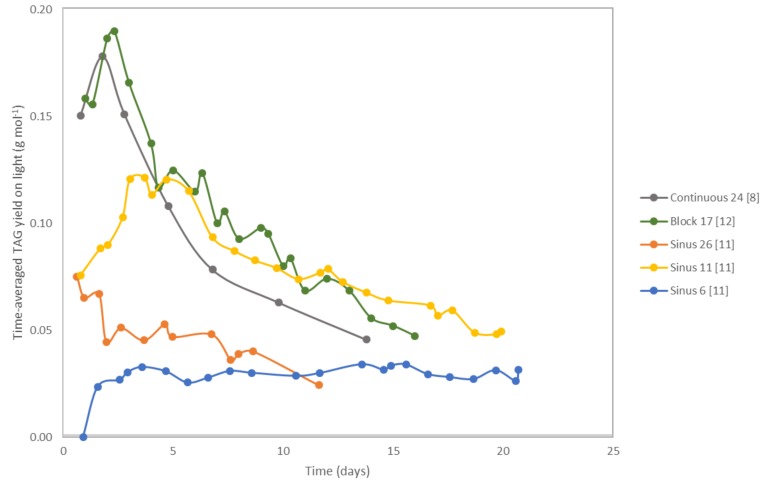
Time-averaged TAG yield on light (g_TAG_ mol_ph_^−1^) for the different biomass-specific photon supply rates and light regimes from the moment of nitrogen starvation [8,11,12].

The maximal time-averaged TAG yield on light reached for the different biomass-specific photon supply rates during nitrogen starvation is shown in Figure 2. The maximal time-averaged TAG yield on light changes over time for the different experiments are shown in Figure A1.

The highest maximal time-averaged TAG yield on light was reached at a biomass-specific photon supply rate between 17 to 24 µmol g_dw_^−1^ s^−1^, when the light intensity was constant and provided either continuously or as a day:night cycle (Figure 2, circles and diamonds). The TAG yields on light achieved under sinus light (Figure 2, triangles) were lower compared to the other experiments. Even though similar biomass-specific photon supply rates were used (24 and 26 µmol g_dw_^−1^ s^−1^), the maximal time-averaged TAG yield on light achieved was different, with 0.18 compared to 0.07 g_TAG_ mol_ph_^−1^ for continuous and sinus light conditions, respectively. Besides the biomass-specific photon supply rate, the light regime also influenced the TAG yield. The lower yield for the sinus light conditions might be the result of a high peak in light intensity (1500 µmol m^−2^ s^−1^) which can cause photosaturation and photoinhibition, resulting in energy waste as heat and damage to the photosystem, and loss of light going through the reactor without being absorbed [11]. It should be noted that the TAG content for the sinus light conditions was higher at the start of the nitrogen starvation compared to continuous light (0.13 compared to 0.08 g_TAG_ g_dw_^−1^). This indicates that stress was experienced before nitrogen starvation in this culture, probably as a result of the high peak in light intensities. 

Since a lower average light intensity was used in the experiment with constant light intensity applied as a day:night cycle, no conclusive statement can be made regarding the effect of constant light intensity applied as a day:night cycle compared to a continuous or sinus light. To distinguish the effect of a lower biomass-specific photon supply rate from the effect of the day:night light regime, an experiment with the same biomass-specific photon supply rate as provided with continuous light (24 µmol g_dw_^−1^ s^−1^) should be used, and the nitrogen starvation should be started by washing the culture. This higher biomass-specific photon supply rate can be achieved by keeping the same incident light intensity and lowering the initial biomass concentration, or by keeping the same initial biomass concentration and increasing the incident light intensity. 

**Figure 3:**
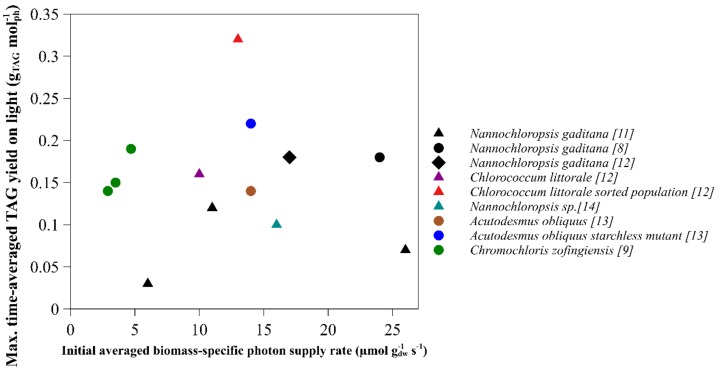
Maximal time-average TAG yield on light comparison for various microalgal species under various initial biomass-specific photon supply rates. The light regime used: continuous (circles), sinus (triangles) and block (diamonds). The yield for *Chromochloris zofingiensis* was calculated based on absorbed light. Details of the calculated biomass-specific photon supply rates from different studies are shown in Table A1 [8,10,11,12,13,14,15].

The achieved maximal time-averaged TAG yields on light were compared to other microalgal species using different biomass-specific photon supply rates and light regimes (Figure 3). The growth conditions, initial biomass concentration, light intensity, light regime and the resulting biomass-specific photon supply rates for the different species with their references are given in Table A1.

No clear trend was found in the maximal time-averaged TAG yields on light for different microalgal species subjected to different biomass-specific photon supply rates at the onset of nitrogen starvation. The TAG yield on light during nitrogen starvation was not only dependent on the biomass-specific photon supply rate and light regime used, but also on the microalgal species used. This should be considered for TAG optimization. The sorted population of *Chlorococcum littorale* showed the highest maximal time-averaged TAG yield on light, indicating that cell sorting could also be an interesting approach for *Nannochloropsis gaditana* to improve the TAG yield [13]. 

### 2.2. TAG Content

**Figure 4:**
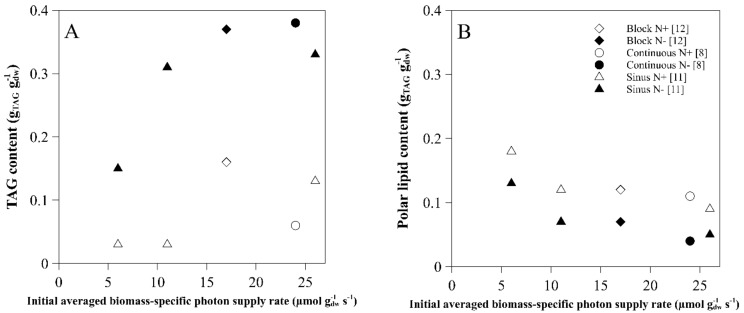
TAG (**A**) and polar lipid (**B**) content (g_TAG_ g_dw_^−1^) at the start of nitrogen starvation (white) and after 14 days (12 days for 26 µmol g_dw_^−1^ s^−1^) of nitrogen starvation (black) for the different average biomass-specific photon supply rates (µmol g_dw_^−1^ s^−1^) at the onset of nitrogen starvation. The light regime used: continuous (circles), sinus (triangles) and block (diamonds) [8,11,12].

The TAG content expressed per dry weight was measured both at the start of the nitrogen starvation and after 14 days of nitrogen starvation for all the different biomass-specific photon supply rates and light regimes used (Figure 4A) [8,11,12]. 

The highest TAG contents expressed per dry weight (0.39 g_TAG_ g_dw_^−1^) was reached at 17 µmol g_dw_^−1^ s^−1^, but similar maximal TAG contents were reached for most biomass-specific photon supply rates. This suggests that there might be a limit to the amount of TAG that can be accumulated in the cell. A minimum cell volume might be necessary for basic cell functioning, resulting in a maximum volume of TAG that can accumulate. There is no current knowledge on this, but it might be an important factor to consider in order to increase lipid productivity, alongside the determination of the maximum cell diameter that can be obtained. If there is a maximal volume in the cell for TAG accumulation, it can be advantageous to have cell division in the first days after nitrogen starvation, or have a (mutant) strain that can produce TAG without impairing growth [16]. Other possibilities are selecting for larger cells with a relatively lower minimum required cell volume for basic cell functioning, or selecting cells with higher TAG productivity [13]. *Nannochloropsis gaditana* still divided on average 1.5 times in the first days after nitrogen starvation.

A much lower TAG content (0.19 g_TAG_ g_dw_^−1^) and consequently a lower TAG yield on light was reached with a biomass-specific light supply rate of 6 µmol g_dw_^−1^ s^−1^. Due to the low biomass-specific photon supply rate, there might have been insufficient energy to induce large TAG accumulation. This could have been caused by a low incident light intensity or by high volumetric maintenance energy requirements due to the high biomass concentration. To study whether energy is indeed limiting, extra energy could be added during the nitrogen starvation phase by increasing light intensity. If this results in increased TAG accumulation, the light was limiting. 

## 3. Polar Lipids

TAG can be synthetized de novo and via the conversion of membrane lipids, which consist mainly of polar lipids. Therefore the effect of the light conditions on the polar lipid content was studied (Figure 4B). In all cultures, the polar lipid content expressed per dry weight decreased from the moment nitrogen starvation commenced [8,11,12]. The polar lipid content at the start of the nitrogen starvation phase was negatively correlated with the biomass-specific photon supply rate; the lowest biomass-specific photon supply rate resulted in the highest polar lipid content. This is probably the result of photoacclimation, the process by which the photosystem increases and possibly polar membrane lipids are created, due to the low light conditions [11]. The polar lipids decreased less than the increase in TAG, indicating that part of the TAG was made de novo. 

## 4. Eicosapentaenoic Acid (EPA) 

**Figure 5:**
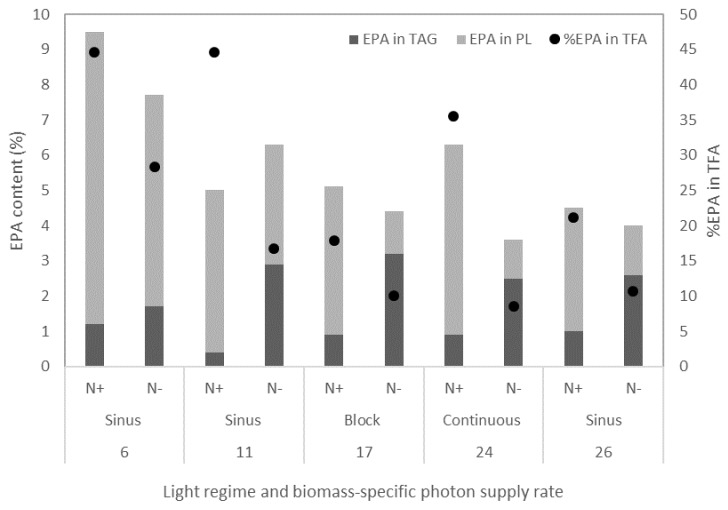
EPA content in TAG and polar lipids (PL) expressed as a percentage of dry weight and the EPA percentage of the total fatty acids (TFA) for the different initial biomass-specific photon supply rates and light regimes. Results are shown at the start of the nitrogen starvation phase (N+) and after 14 days of nitrogen starvation (12 days for 26 µmol g_dw_^−1^ s^−1^) (N−) [8,11,12].

**Figure A2:**
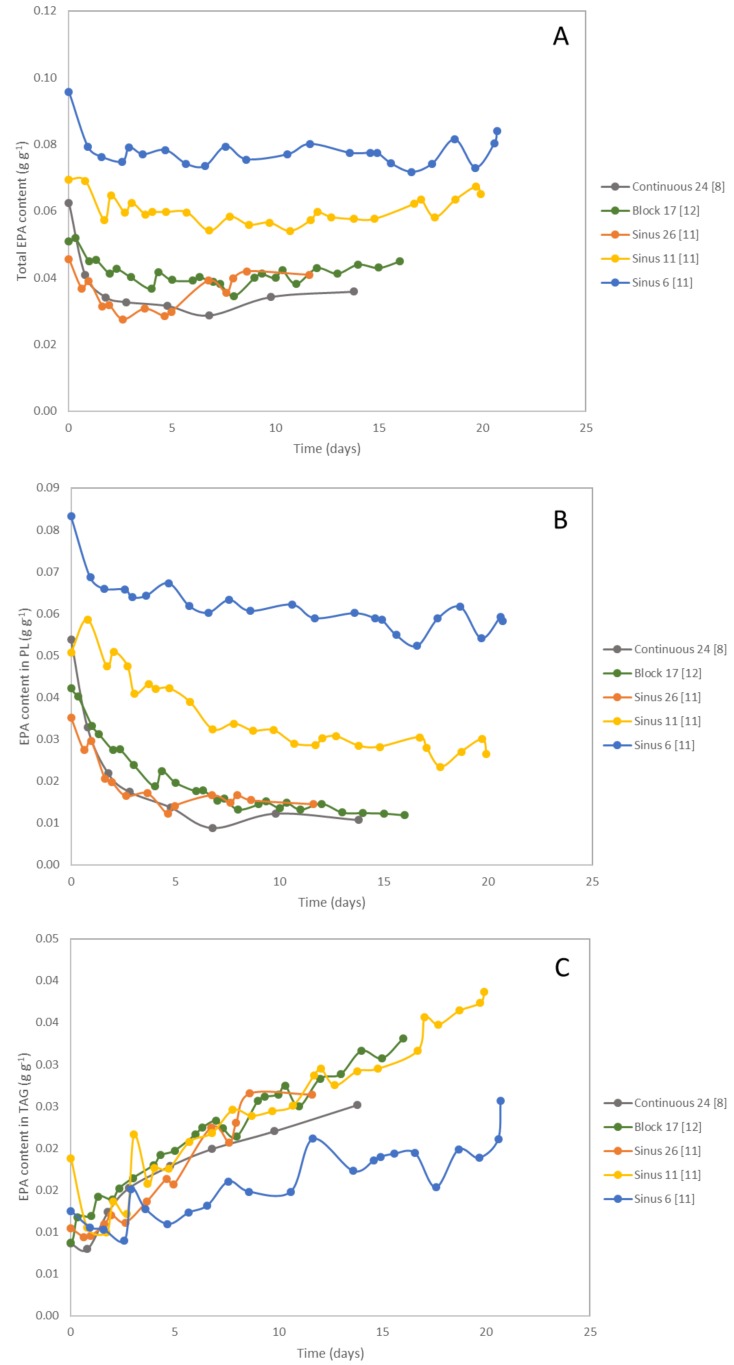
EPA content expressed per biomass (g_EPA_ g_dw_^−1^) in total lipids (TAG + PL) (**A**), in TAG (**B**) and in polar lipids (PL) (**C**) for the different biomass-specific photon supply rates and light regimes from the moment of nitrogen starvation [8,11,12].

The fatty acid EPA accumulated both in the polar lipids in the membranes and in the TAG lipid bodies. Figure 5 shows the EPA contents expressed per dry weights in both lipid fractions both at the start and after 14 days of nitrogen starvation. The change of the EPA content expressed per dry weight in the TAG, polar lipids and in the total lipids (TAG + PL) for all days during nitrogen starvation is shown in Figure A2. 

The highest EPA content was reached with the lowest biomass-specific photon supply rate at the onset of nitrogen starvation. Similar to our results, higher percentages of EPA were found for *Nannochloropsi*s sp. cultivated under low light compared to high light [17]. This high EPA content was mainly due to the EPA present in polar lipids. At low biomass-specific photon supply rates, more membranes are formed as a result of photoacclimation, leading to higher polar lipids and consequently EPA contents in the cell. This was also found for *Nannochloropsis* sp. cultivated under low light conditions [18]. The high EPA content at the start of the nitrogen starvation phase is interesting for EPA production, however, it should be noted that the growth phase took much longer to obtain a high biomass concentration compared to other experiments (21 days compared to 7 to 11 days). 

**Figure A3:**
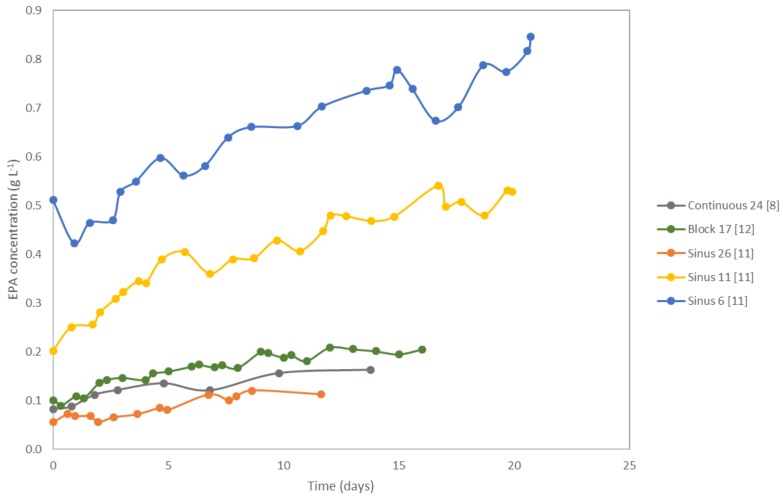
Total EPA concentration (g L^−1^) measured in the photobioreactor for the different biomass-specific photon supply rates and light regimes from the moment of nitrogen starvation [8,11,12].

The total EPA content expressed per dry weight decreased slightly in most experiments during nitrogen starvation. As a result of the increased biomass concentration, however, the total EPA concentration in the reactor increased (Figure A3). This shows that part of the EPA has to be produced de novo during nitrogen starvation, as confirmed in the labelling study in [8]. In all experiments, the EPA percentage of total fatty acids (TFA) decreased during nitrogen starvation due to the faster increase of other fatty acids in TAG, mainly palmitic acid and palmitoleic acid. 

**Figure A4:**
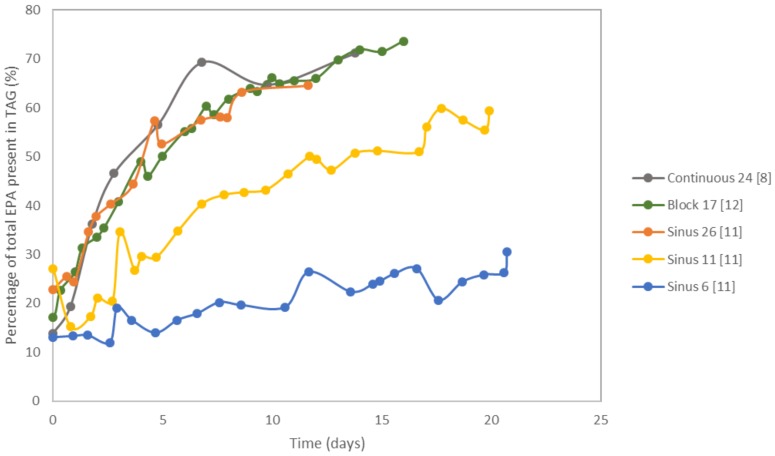
The percentage of all EPA in the cell, which is present in TAG lipid fraction for the different biomass-specific photon supply rates and light regimes [8,11,12].

The distribution of EPA in the cell showed that the EPA content in TAG was similar for all cultures at the start of nitrogen starvation (Figure 5, N+ bars). During nitrogen starvation, the EPA content in the TAG increased, and after 14 days of nitrogen starvation a large fraction of EPA in the cell was present in the TAG lipid fraction. For most conditions, the fraction of EPA in the cell present in the TAG was 60 to 70%, except for the cultures with the lowest biomass-specific photon supply rates (sinus light 11 and 6 µmol g_dw_^−1^ s^−1^) which were 50% and 25%, respectively (Figure A4). One possible strategy to increase the amount of EPA in the cell during nitrogen starvation is by increasing the amount of EPA accumulated in the TAG.

A better understanding of the function of EPA accumulation can help with finding targets to increase its accumulation. It is hypothesized that EPA accumulation in TAG acts as storage for membrane components to be rapidly used upon better growth conditions. In *Parietochloris incisa,* a radiolabeling study showed that the arachidonic acid present in TAG was used for chloroplast membrane lipid upon nitrogen recovery [19]. To conclude whether EPA is stored in TAG by *Nannochloropsis gaditana* during nitrogen starvation for membrane synthesis upon nitrogen recovery, a labelling experiment similar to [8] should be carried out. An initial growth phase should be implemented, followed by nitrogen starvation with labelled carbon (^13^C) and concluded by a third phase in which nitrogen is re-supplied and the carbon source is changed to ^12^C carbon. The newly synthesized fatty acids and the fatty acids that translocated from the TAG to the membranes during the recovery phase can then be distinguished by measuring the ^12^C and ^13^C fatty acids. This will provide an answer to the hypothesis that TAG acts as a storage for plastid material during nitrogen starvation. 

Previously, it was shown that 23% of the EPA accumulated in TAG was intact translocated from the polar lipids during nitrogen starvation, and 21% of the EPA accumulated in TAG was completely made de novo [8]. Since de novo synthesis, translocation and carbon recycling cannot be distinguished using only fatty acids data, no conclusions can be drawn on its distribution under different biomass-specific photon supply rates. Both translocation and de novo synthesis are important for EPA accumulation in TAG. Possible limiting factors for the accumulation of EPA in TAG, such as the EPA content in membranes at the start of nitrogen starvation, de novo synthesis, and translocation will be discussed.

The amount of EPA in polar lipids could be a limiting factor determining translocation into TAG. The lowest biomass-specific photon absorption rate had, however, the highest EPA and polar lipid content but the lowest fraction of EPA present in TAG. This suggests that the EPA accumulation in TAG is not limited by the amount of EPA present in the polar lipids. The culture with the lowest biomass-specific photon supply rate has the lowest TAG content. Therefore, EPA accumulation in TAG might be correlated to TAG content and productivity. 

Another hypothesis explaining the limited EPA accumulation in TAG could be the maximal amount of EPA that can accumulate in TAG without steric hindrance. In *Trachydiscus minutus*, a species from the same class as *Nannochloropsis*, TAG containing three EPA acyl groups was found [20]. 

The translocation of EPA might be limited by the enzymes involved in translocation. Two types of enzymes that might be involved in the translocation of fatty acids from membranes to TAG are phospholipid:diacylglycerol transferase (PDAT) and lipases [19]. Knock-out studies of these enzymes might provide more insight into their effect on EPA accumulation in TAG during nitrogen starvation, and allow the specification of the enzyme(s) that are most important for the translocation of EPA in *Nannochloropsis gaditana* [21]. The overexpression of these enzymes might increase the translocation of EPA into TAG. Measuring the expression levels of PDAT and lipases under conditions with increased or decreased EPA accumulation in TAG might also give an indication of the specific genes responsible for translocation. 

Furthermore, it would be interesting to study the substrate specificity of, for example, acyltransferases, since EPA seems to be translocated with higher affinity compared to other fatty acids [8,21]. 

Finally, de novo synthesis can be rate limiting for EPA accumulation in TAG. If an overexpression of the enzymes responsible for de novo EPA synthesis (e.g., elongases and desaturases) increases EPA synthesis, measuring the localization of EPA in TAG or polar lipids will help understand EPA accumulation in TAG during nitrogen starvation. 

## 5. Conclusions

Most light regimes and biomass-specific photon supply rates at the onset of nitrogen starvation result in similar high TAG contents and thus do not seem to affect the TAG content. The EPA content was highest at the lowest biomass-specific photon supply rate, mainly due to its presence in the polar lipids which are mostly located in the membranes. During nitrogen starvation, the EPA accumulation in TAG was lower at the low biomass-specific photon supply rates, which had the highest EPA content in the polar lipids. This suggests that the amount of EPA in the polar lipids is not limiting for the EPA accumulation in TAG. More research is necessary to understand the mechanisms and regulations involved in EPA accumulation during nitrogen starvation, and its localization in polar lipids and TAG. A study is suggested of the function of EPA in TAG using a nitrogen recovery experiment with labelled carbon (^13^C). One of the possibilities to increase EPA accumulation during nitrogen starvation is to increase its accumulation in TAG. Knock-out studies of PDAT and lipase genes can indicate which enzyme(s) are responsible for the translocation of EPA. These genes are possible targets for overexpression in order to increase the translocation of EPA into TAG. Besides increased translocation, de novo synthesis could be increased through the overexpression of elongases and desaturases involved in the EPA synthesis pathway. This shows that there are several future opportunities to study and increase EPA accumulation.

## Appendix A

**Table A1:** Comparison TAG yield on light of different microalgae species using different biomass-specific photon supply rates and their respective references.

Species	Light Regime	Initial Biomass Concentration (g L^−1^)	Average Light Intensity per day (µmol m^−2^ s^−1^)	Initial Average Biomass-Specific Photon Supply Rate (µmol g^−1^ s^−1^)	TAG Yield on Light (g mol^−1^)	Reference	Remarks
*Nannochloropsis gaditana*	Continuous	1.3	636	24	0.18	[8]	
*Nannochloropsis gaditana*	Block	1.2	424	17	0.19	[12]	Runout estimated by nitrogen content
*Nannochloropsis gaditana*	Sinus	1.2	636	26	0.07	[11]	
*Nannochloropsis gaditana*	Sinus	2.9	636	11	0.12	[11]	
*Nannochloropsis gaditana*	Sinus	5.4	636	6	0.03	[11]	
*Nannochloropsis* sp.	Sinus	1.9	636	16	0.1	[15]	
*Chlorococcum littorale*	Sinus	3.1	636	10	0.16	[13]	
*Chlorococcum littorale* (sorted)	Sinus	2.4	636	13	0.32	[13]	
*Chromochloris zofingiensis*	Continuous	2.5	245	4.7	0.19	[10]	Yield absorbed light
*Chromochloris zofingiensis*	Continuous	3.4	245	3.5	0.15	[10]	
*Chromochloris zofingiensis*	Continuous	4.1	245	2.9	0.14	[10]	
*Acutodesmus obliquus10*	Continuous	1.7	500	14	0.14	[14]	Runout between 1.5–2 g/L averaged
*Acutodesmus obliquus* (starchless mutant)	Continuous	1.7	500	14	0.22	[14]	Runout between 1.5–2 g/L averaged
